# Ginkgolic Acid Protects against Aβ-Induced Synaptic Dysfunction in the Hippocampus

**DOI:** 10.3389/fphar.2016.00401

**Published:** 2016-10-26

**Authors:** Dalila Mango, Filippo Weisz, Robert Nisticò

**Affiliations:** ^1^Department of Physiology and Pharmacology, University of Rome “Sapienza”Rome, Italy; ^2^European Brain Research Institute, Rita Levi-Montalcini FoundationRome, Italy; ^3^Department of Biology, University of Rome Tor VergataRome, Italy

**Keywords:** ginkgolic acid, hippocampus, LTP, Aβ, electrophysiology

## Abstract

*Ginkgo* leaf is the most used form of supplement for cognitive ailments. The standardized extract formulation EGb 761 is a dietary supplement with proven benefit in several neurological and psychiatric conditions including memory decline in Alzheimer’s disease, schizophrenia and dementia. Ginkgolic acid (GA) is a component of this extract which shows pleiotropic effects including antitumoral and anti-HIV action; however, its effect on memory is still unknown. Here, we carried out an electrophysiological analysis to investigate the effects of GA on long term potentiation and synaptic transmission at CA1 hippocampal synapses. We also evaluated the potential rescuing effect of GA on the synaptic dysfunction following *in vitro* application of Aβ. Data obtained indicate that GA exerts neuroprotective effects against Aβ-induced impairment of neurotransmitter release and synaptic plasticity.

## Introduction

*Ginkgo biloba* L. (Mantissa Plantarum Altera, 1771, Ginkgoaceae) leaves and the nuts have been in use for several centuries in traditional Chinese medicine. However, it was only in the last 20–30 years that the use of *Ginkgo* leaf and its standardized extract formulation (EGb 761) has been widely used as a form of supplement for cognitive ailments (Mahadevan and Park, 2008). *Ginkgo* leaf extract has shown beneficial effect in different pathologies such as Alzheimer’s disease (AD), memory loss, cardiovascular disease, cancer, age-related macular degeneration, and psychiatric disorders like schizophrenia (Gessner et al., 1985; Kennedy et al., 2007; Ramassamy et al., 2007; Scripnikov et al., 2007). Ginkgolic acid (GA) is a component of EGb 761, currently used as memory enhancer and as a dietary supplement with proven benefit in several neurological and psychiatric conditions (Le Bars et al., 1997; Hsu et al., 1999). Nonetheless, because EGb 761 is a complex combination of several potentially active components that may be acting synergistically, the exact mechanism of action remains difficult to elucidate. AD is a neurodegenerative disorder characterized by the progressive loss of neurons, deposition of insoluble aggregates of two proteins in the brain, amyloid-β (Aβ), and the microtubule associated protein tau (MAPT). Synaptic deterioration occurs early in the disease, well before the formation of amyloid plaques and neuron loss (Thompson et al., 2003; Arendt, 2009). This early synaptic impairment is evident in different brain areas, including the hippocampus, and entorhinal cortex, which are involved in cognitive process and memory formation (Thompson et al., 2003; Arendt, 2009).

Ginkgolic acid has controversial effects, which were characterized in several studies (Ude et al., 2013). Indeed, GA is able to induce neuronal death at high concentrations (Ahlemeyer et al., 2001); accordingly, today all commercial preparations of *Ginkgo* leaf contain low dose of GA in order to minimize side effects (McKenna et al., 2001). However, there is recent evidence that GA has different protective effects, as well as antitumoral and anti-HIV effects (Wang H. et al., 2010; Zhou et al., 2010; Lü et al., 2012).

To shed same light on the potential effects of GA on memory, we carried out an electrophysiological study analyzing long term potentiation (LTP), a molecular mechanism which underlies learning and memory (Bliss and Collingridge, 1993), and excitatory synaptic transmission in the *in vitro* hippocampus. Also, we investigated the protective effects of GA against Aβ-induced synaptic impairment (Klyubin et al., 2008; Olsen and Sheng, 2012; Yao et al., 2013; Varga et al., 2015).

## Materials and Methods

### Slices Preparation

All experiments followed international guidelines on the ethical use of animals from the European Communities (Santa Lucia Foundation, Rome, Italy) Council Directive 2010/64/EU. C57BL6 mice (30–40 days old) were deeply anesthetized with halothane and killed by decapitation. The brain was rapidly removed from the skull and parasagittal hippocampal slices (250 μm thick) were cut with a vibratome (VT 1200S, Leica) in cold (0°C) artificial cerebrospinal fluid (aCSF) containing (in mM): NaCl 124; KCl 3; MgSO4 1; CaCl2 2; NaH2PO4 1.25; NaHCO3 26; glucose 10; saturated with 95% O2, 5% CO2 (pH 7.4; Cold Spring Harbor Protocols), and left to recover for 1 h in ACSF at room temperature.

### Whole-Cell Patch Clamp Recordings

Individual slices were placed in a recording chamber, on the stage of an upright microscope (Zeiss, Germany) and submerged in a continuously flowing (3 ml/min) solution at 30°C (±2°C). Individual neurons were visualized through a 40× water-immersion objective (Olympus, Japan) connected to infrared video microscopy (Hamamatsu, Japan). Borosilicate glass electrodes (5–7 MΩ), pulled with a PP 83 Narishige puller, were filled with a solution containing the following (in mM): CsCH3SO3 115; CsCl 10; KCl 10; CaCl2 0.45; EGTA 1; Hepes 10; QX-314 5; Na3-GTP 0.3; Mg-ATP 4.0; pH adjusted to 7.3 pH with CsOH. Whole-cell voltage clamp (at -70 mV holding potential) or current clamp experiments were carried out with a MultiClamp 700B amplifier (Axon Instruments, Foster City, CA, USA), filtered at 1 kHz and digitized (5 kHz).

Excitatory post-synaptic currents (EPSCs) were elicited by monopolar stimulating electrode placed in *stratum radiatum* in order to stimulate Schaffer collateral fibers. LTP was induced by 30 pulses (0 mV holding potential) as previously described (Peineau et al., 2007).

Some slices were incubated for 30 min with GA (1–30 μM) or Aβ1–42 (Aβ 200–500 nM) or both before recordings; other recordings (control condition) were performed from non-incubated slices. All experiments were made in the presence of the GABA antagonist picrotoxin in order to pharmacologically isolate the EPSC.

For paired-pulse ratio (PPR) experiments, paired-pulse stimuli (50 ms inter-pulse interval) were elicited with stimulating electrode placed close to the recording neuron. Spontaneous excitatory post-synaptic currents (sEPSCs) were recorded in the presence of picrotoxin (100 μM).

### Statistical Analysis

Excitatory post-synaptic currents peak amplitude was normalized to baseline; statistical significance was evaluated by paired or unpaired Student’s *t*-test, according to the groups compared, between 50 and 60 min following delivery of conditioning trains. The PPR was calculated as the ratio of the second EPSC amplitude to the first. Amplitude and frequency of sEPSC was evaluated on 3 min recordings. All values were described as mean ± SEM. Statistical significance was set at *p* < 0.05. For all statistical comparisons, the *n* used reflected the number of neurons recorded.

### Drugs

Picrotoxin and GA was purchased from Abcam (Milan, Italy); Aβ1–42 was purchased from Sigma-Aldrich. When drugs were dissolved in DMSO, the final concentration of DMSO did not exceed 0.5%.

## Results

### Ginkgolic Acid Enhanced Hippocampal LTP

Whole-cell recordings were obtained from hippocampal CA1 pyramidal neurons and synaptic responses were evoked by electrical stimulation of Schaffer collateral afferents using a monopolar electrode. To induce LTP, the stimulating train was applied 5 min after the whole cell configuration, as previously described (Peineau et al., 2007).

**FIGURE 1:**
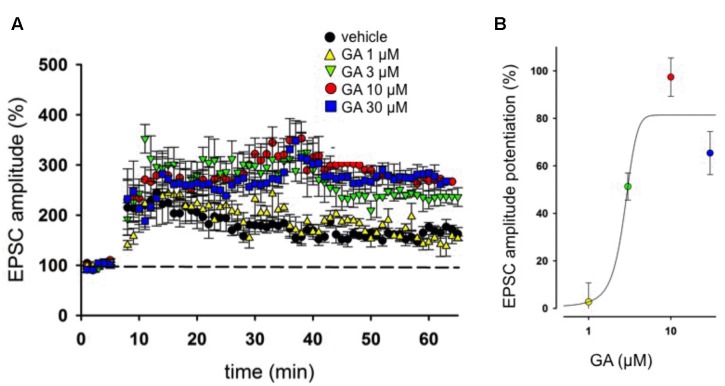
**Ginkgolic acid (GA) modulates long term potentiation (LTP) in hippocampus.** **(A)** Superimposed pooled data showing the effect of GA (1–30 μM) on LTP compared to control. **(B)** Concentration-response curve showing the effects of GA on the Excitatory post-synaptic currents (EPSC; measured as % of control) 1 h after induction of LTP. Each plot represents the recording of at least six separate neurons.

In our experimental condition, in nine control neurons the LTP elicited was 164 ± 11%. In neurons from slices incubated with GA, we found a dose-dependent increase in LTP magnitude compared to control condition. Indeed, GA, at the concentration of 1 μM, did not affect LTP magnitude (155 ± 13%, *n* = 6, *p* > 0.05, **Figures 1A,B**). On the other hand, treatment with GA between 3 and 30 μM was able to increase significantly LTP compared to vehicle (3 μM, 237 ± 14%, *n* = 6; 10 μM, 270 ± 15%, *n* = 7; 30 μM, 275 ± 11%, *n* = 8, *p* < 0.001, **Figures 1A,B**).

### Ginkgolic Acid Restores LTP Impairment Following Aβ Application

**FIGURE 2:**
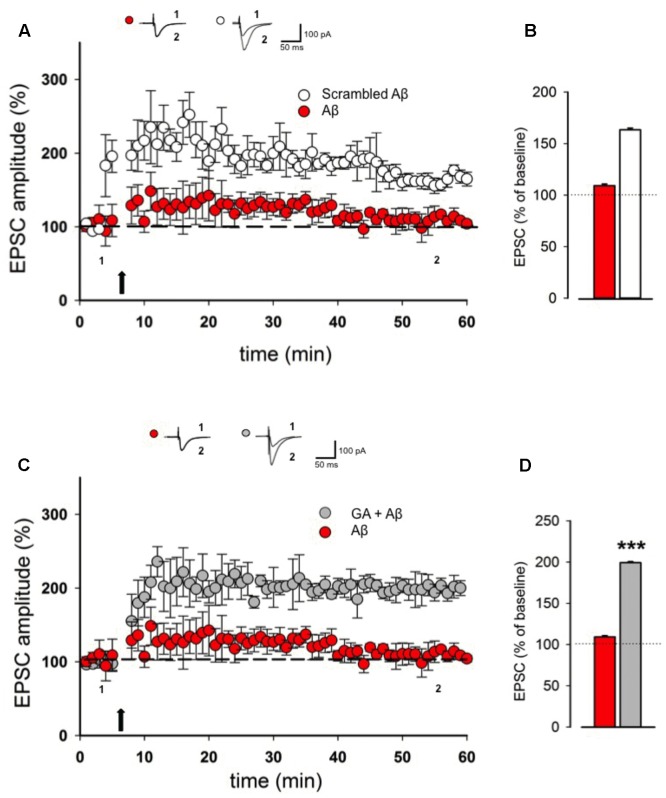
**Ginkgolic acid reverses Aβ-mediated LTP impairment.** **(A)** Superimposed pooled data showing LTP in slices treated with Aβ or scrambled Aβ. On top, representative traces for both conditions are shown. **(B)** Histograms illustrating the magnitude of LTP (% of baseline) in the two experimental conditions. **(C)** Superimposed pooled data showing LTP in slices treated with Aβ or GA + Aβ. On top, representative traces for both conditions are shown. **(D)** Histograms illustrating the magnitude of LTP (% of baseline) in the two experimental conditions. ^∗∗∗^*p* < 0.001.

It is known that Aβ oligomers are able to perturb hippocampal LTP (Chapman et al., 1999; Chen et al., 2000; Varga et al., 2015) and were also reported to alter synaptic glutamate (Glu)-recycling and transmission (Yao et al., 2013; Varga et al., 2015). Here we wanted to test whether GA exerts a neuroprotective action against Aβ-induced LTP deficiency. In our experimental system, pretreatment of slices for 30 min with 200 nM of Aβ was able to fully block LTP in hippocampal CA1 pyramidal neurons (109 ± 10%, *n* = 7, *p* > 0.05, **Figures 2A,B**). This effect did not occur when slices were pretreated with scrambled form of Aβ (157 ± 8%, *n* = 5, *p* < 0.001, **Figures 2A,B**). Notably, co-application of GA (1 μM) was able to reverse LTP impairment induced by Aβ (199 ± 14%, *n* = 5, *p* < 0.001, **Figures 2C,D**).

### Ginkgolic Acid Restores PPR Alteration Following Aβ Application

**FIGURE 3:**
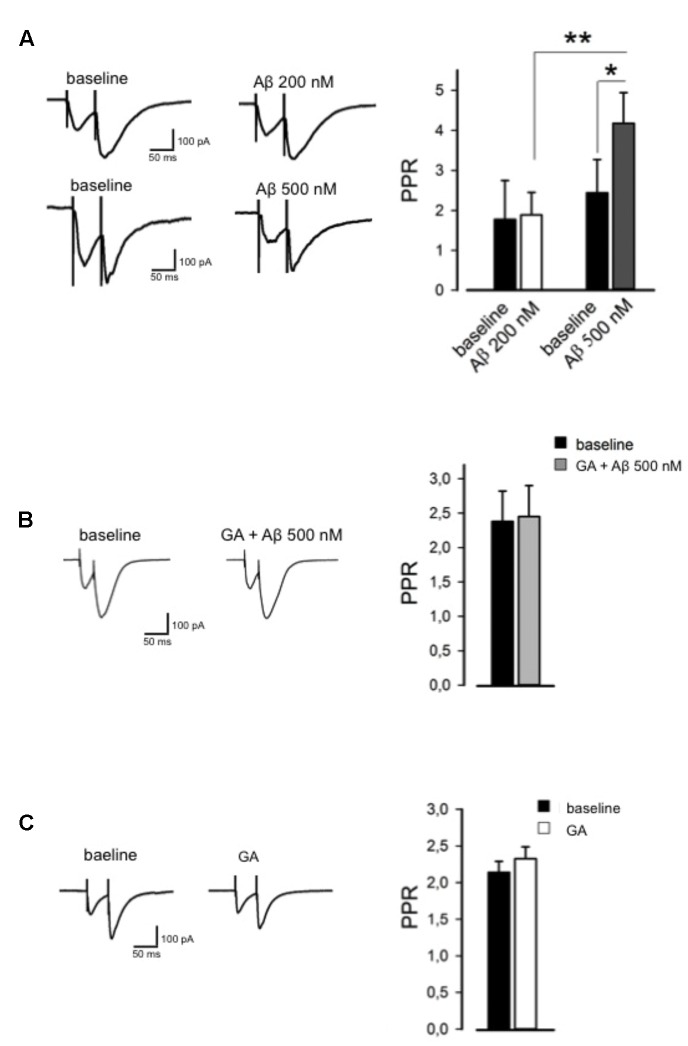
**Ginkgolic acid reverses Aβ-mediated paired-pulse ratio (PPR) alteration.** **(A)** On the left, representative traces are shown; on the right, histograms show change in PPR. **(B)** GA reverses Aβ-induced change in PPR. On the left, representative traces in baseline and GA+Aβ conditions are shown. On the right, histograms show PPR in both conditions. **(C)** GA alone did not alter PPR. On the left, representative traces are shown; on the right, histograms show no change in PPR. ^∗^*p* < 0.05; ^∗∗^*p* < 0.01.

To test whether Aβ influences basal synaptic transmission, we recorded evoked EPSCs of hippocampal CA1 pyramidal neurons. After a stable baseline was obtained, we perfused Aβ (200 nM, 30 min) and found it had no effect on basal synaptic transmission (98 ± 6%, *n* = 6, *p* > 0.05). However, when applied at 500 nM, Aβ caused a marked depression in EPSC amplitude when compared to baseline (53 ± 7%, *n* = 7, *p* < 0.01). We then studied paired pulse facilitation (PPF) paradigm, which represents a presynaptic form of synaptic plasticity. Perfusion of 200 nM Aβ did not alter the PPR, whereas 500 nM Aβ induced a significant increase in the PPR (4 ± 0.8, *n* = 7, *p* < 0.05, **Figure 3A**) compared to baseline (2 ± 0.9, **Figure 3B**), which is associated with a reduced release of neurotransmitter. Pretreatment with GA (1 μM) before Aβ application (500 nM) normalized the PPF alteration to the baseline level (*n* = 7, *p* > 0.05, **Figure 3B**), while GA *per se* did not affect PPR (*n* = 5, *p* > 0.05, **Figure 3C**).

### Ginkgolic Acid Rescues Aβ-mediated Effects on Excitatory Transmission

**FIGURE 4:**
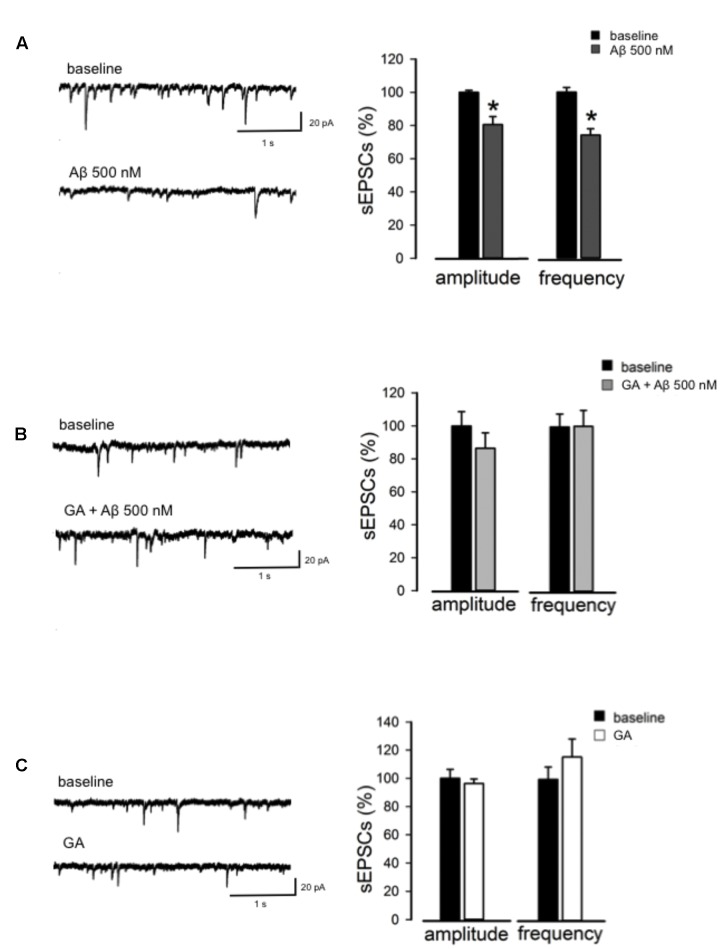
**GA normalizes Aβ-mediated change in excitatory neurotransmission.** **(A)** Reduction in amplitude and frequency of Spontaneous excitatory post-synaptic currents (sEPSC) following Aβ perfusion. On the left, representative traces are shown; on the right, histograms show the effect of Aβ on amplitude and frequency of sEPSC (as % of baseline). **(B)** GA prevented Aβ-triggered impairment of sEPSC. On the left, traces of sEPSC are shown. On the right, histograms show effect of GA+Aβ on amplitude and frequency of sEPSC (as % of baseline). **(C)** GA alone did not alter sEPSC amplitude and frequency. On the left, representative traces in baseline conditions and following perfusion of GA are shown. On the right, histograms show effect of GA on amplitude and frequency of sEPSC (as % of baseline). ^∗^*p* < 0.05.

Next, we studied synaptic transmission by recording sEPSC from CA1 pyramidal neurons. We found that 500 nM Aβ significantly suppressed both sEPSCs amplitude (80 ± 4%, *n* = 8, *p* < 0.05, **Figure 4A**) and frequency (74 ± 4%, *n* = 8, *p* < 0.05, **Figure 4A**) compared to baseline, suggesting that Aβ-induced inhibition of synaptic transmission relies on both presynaptic and postsynaptic mechanisms. Next, we investigated whether GA was able to reverse the Aβ-mediated effect on excitatory synaptic transmission. In the same conditions, GA was able to reverse the changes induced by Aβ on both the amplitude and frequency of sEPSC (*n* = 6, *p* > 0.05, **Figure 4B**). Notably, GA alone neither influenced the amplitude nor the frequency of sEPSC (*n* = 6, *p* > 0.05, **Figure 4C**).

## Discussion

The present study demonstrates, for the first time, that GA, the major component of *Gingko biloba* standardized extract formulation EGb 761, rescues Aβ-mediated alteration of LTP, PPF, and spontaneous excitatory transmission in CA1 hippocampal pyramidal neurons. In line with this, previous studies have demonstrated that EGb 761 component acts as memory enhancer thus improving cognitive decline in AD patients (Amieva et al., 2013), as well as LTP and hippocampal-dependent working memory following acute perfusion or chronic treatment, respectively (Williams et al., 2004; Wang et al., 2006).

It has been established that nanomolar concentrations of Aβ cause inhibition of LTP mainly through postsynaptic mechanisms, whereas keeping basal synaptic transmission intact (Vitolo et al., 2002; Puzzo et al., 2005; Monfort and Felipo, 2010; Wang X.H. et al., 2010). On the other hand, presynaptic dysfunction has only been observed following the perfusion of high nanomolar to low micromolar concentrations of Aβ (Santos-Torres et al., 2007; Yao et al., 2013). To test neuroprotective action of GA against Aβ, we chose the concentration at which GA *per se* does not affect synaptic plasticity.

Interestingly, here we show that GA was able to rescue Aβ-mediated LTP impairment at a concentration ineffective to affect basal neurotransmission and LTP. In addition, our results demonstrate that GA could reverse Aβ-induced alterations of either PPR or sEPSC, which measure both pre- and post-synaptic function, respectively. We have not investigated so far the mechanism underlying GA effect. Our results might suggest that both pre- and post-synaptic mechanisms might be involved in its neuroprotective action.

Based on the current literature, putative molecular targets might involve SUMOylation, a post-translational modification that is known to control many aspects of cell function also at the neuronal level (Martin et al., 2007; Henley et al., 2014). In particular, it has been demonstrated that SUMOylation is among the mechanisms that links Aβ to synaptic dysfunction (Fukuda et al., 2009; Lee et al., 2013, 2014, Nisticò et al., 2014). Indeed, GA directly binds E1 and inhibits the formation of E1-SUMO intermediate at the same range of concentrations here used to prevent LTP inhibition by Aβ (Fukuda et al., 2009). In addition, GA can also inhibit the SUMOylation of p53 (Fukuda et al., 2009), which is known to play a crucial role in the early phase of LTP (Pustylnyak et al., 2015) and has been shown to be involved in aging and AD (LaFerla et al., 1996; Lanni et al., 2012).

Another possible mechanism might rely on the Bcl-2/Bax pathway, which contributes to the antitumoral and anti-HIV effects of GA (Wang H. et al., 2010; Zhou et al., 2010; Lü et al., 2012). Inasmuch, as Bcl-2/Bax is also involved in Aβ-mediated impairment of LTP (Olsen and Sheng, 2012), it can be hypothesized that the protective effect of GA here observed might be mediated by Bcl-2/Bax inhibition.

In addition, a recent work suggested that GA inactivates PI3K/Akt/mTOR in lung cancer cells (Baek et al., 2016). The PI3K/Akt/mTOR is a crucial pathway regulating autophagy and synaptic plasticity in the brain (Heras-Sandoval et al., 2014). Dysregulation of this pathway is commonly reported in brains from AD patients and in AD model mice and was demonstrated to occur by means of Aβ-promoted autophagy (Heras-Sandoval et al., 2014). Thus, inactivation of PI3K/Akt/mTOR pathway by GA might be a protective mechanism against Aβ-promoted autophagy.

To conclude, our work investigated the effect of GA on hippocampal plasticity, neurotransmitter release, and excitatory neurotransmission. In particular, we demonstrate for the first time that GA acts at the synaptic level affording neuroprotection against Aβ-mediated impairment, possibly representing a novel approach to AD prevention and cure.

## Author Contributions

DM designed the research, performed experiments, analyzed data, and wrote the paper, FW analyzed data, RN designed the research, interpreted data, and wrote the paper.

## Conflict of Interest Statement

The authors declare that the research was conducted in the absence of any commercial or financial relationships that could be construed as a potential conflict of interest.
